# Knowledge Levels Regarding Crimean-Congo Hemorrhagic Fever Among Emergency Healthcare Workers in an Endemic Region

**DOI:** 10.14740/jocmr1801w

**Published:** 2014-03-31

**Authors:** Sadiye Yolcu, Cigdem Kader, Afsin Emre Kayipmaz, Sedat Ozbay, Ayse Erbay

**Affiliations:** a Department of Emergency Medicine, Bozok University School of Medicine, Yozgat, Turkey; b Department of Infectious Diseases, Bozok University School of Medicine, Yozgat, Turkey; c Department of Emergency Medicine, Sivas Numune Hospital, Sivas, Turkey

**Keywords:** Crimean-Congo hemorrhagic fever, Emergency, Healthcare worker

## Abstract

**Background:**

In this study, we aimed to determine knowledge levels regarding Crimean-Congo hemorrhagic fever (CCHF) among emergency healthcare workers (HCWs) in an endemic region.

**Methods:**

A questionnaire form consisting of questions about CCHF was applied to the participants.

**Results:**

The mean age was 29.6 ± 6.5 years (range 19 - 45). Fifty-four (49.5%) participants were physicians, 39 (35.8%) were nurses and 16 (14.7%) were paramedics. All of the participants were aware of CCHF, and 48 (44%) of them had previously followed CCHF patients. Rates of the use of protective equipment (masks and gloves) during interventions for patients who were admitted to the emergency service with active hemorrhage were 100% among paramedics, 76.9% among nurses and 61.1% among physicians (P = 0.003). Among 86 (78.9%) HCWs who believed that their knowledge regarding CCHF was adequate, 62 (56.9%) declared that they would prefer not to care for patients with CCHF (P = 0.608).

**Conclusions:**

The use of techniques to prevent transmission of this disease, including gloves, face masks, face visors and box coats, should be explained to emergency room HCWs, and encouragement should be provided for using these techniques.

## Introduction

Crimean-Congo hemorrhagic fever (CCHF) was first described in 1944 [1]. The CCHF virus is transmitted to humans via the bites of infected ticks or by direct contact with the secretions or blood of infected animals or humans. CCHF is a potentially fatal infection. It is endemic in over 30 countries around the Black Sea and in the Middle East and Africa [2]. CCHF infections were first reported in Turkey in 2003 among individuals who became sick in 2002 [3, 4].

In endemic regions, individuals who have occupational contact with livestock and wild animals, including shepherds, farmers and veterinarians, are at high risk for CCHF [5, 6].

Similarly, healthcare workers (HCWs) caring for CCHF patients are the second major group at risk for infection [7]. In Turkey, the neighboring cities of Sivas and Yozgat are endemic sites for CCHF. The climate of these cities is suitable for the survival of ticks, and the first cases of CCHF virus infection in Turkey were reported in this region [3].

HCWs are at risk for blood- and secretion-borne pathogens. Beltrami et al reported that at least 20 pathogens can be transmitted by needle sticks or sharps injuries. These pathogens can be transmitted to HCWs via blood and secretions [8]. Emergency HCWs are also at risk for these infectious diseases [9]. Outbreaks of CCHF among HCWs have been frequently reported and have a high mortality. The highest risk of transmission is from percutaneous exposure [10-14].

In this study, we aimed to determine knowledge levels regarding CCHF among emergency service (ES) HCWs in the cities of Sivas and Yozgat, where CCHF is endemic.

## Materials and Methods

After approval by the local ethics committee, the study was conducted at the Sivas Numune Hospital Emergency Service, the Sivas Government Hospital Emergency Service and the Yozgat Government Hospital Emergency Service. A total of 109 HCWs (54 doctors, 39 nurses and 16 paramedics) received a questionnaire. Data regarding the age, gender and occupation (in the ES) of the participants were recorded. The questionnaire consisted of 28 questions about the workers’ knowledge levels regarding CCHF and their approaches to CCHF (Table 1).

**Table 1:** Questions Asked to Participants

Q1	Have you ever heard of CCHF?
Q2	Do you think that your CCHF knowledge level is sufficient?
Q3	Where did you obtain your CCHF knowledge? a. During my education /b. media/ c. seminars-occupational education/ d. patients whom I followed up.
Q4	Have you ever worked in a clinic where CCHF patients received follow-up care?
Q5	Do you always use gloves during interventions for patients with hemorrhage in the emergency room setting?
Q6	Do you always use protective equipment (masks, gloves, and so on) during interventions for patients who are admitted to the emergency room with nausea, vomiting and enteritis?
Q7	Do you always use protective equipment (masks, gloves, and so on) during interventions for patients who are admitted to the emergency room with active hemorrhage?
Q8	Do you always use gloves during invasive procedures in the emergency room?
Q9	Do you dispose of used sharp equipment and needles in the medical waste box after invasive procedures in the emergency room?
Q10	What is your approach to a suspicious CCHF patient?: a. The patient should be hospitalized immediately/ b. Contact isolation precautions should be implemented/ c. Gloves should be used/ d. Facemasks should be used/ e. Sharp equipment and used needles should be disposed of in a medical waste box/ f. Usage of 1/10 diluted bleach is sufficient to disinfect environments that are contaminated with patients’ blood and secretions.
Q11	Is CCHF a hemorrhagic viral infection?
Q12	Does CCHF occur due to infection of the human body by the CCHF virus?
Q13	Can the CCHF virus be transmitted to humans by ticks?
Q14	Can all ticks carry and transmit CCHF?
Q15	Can the CCHF virus be transmitted from human to human?
Q16	Is CCHF especially common in the summer?
Q17	Can CCHF be an asymptomatic disease?
Q18	Should adhered ticks on the human body be removed by pouring a substance that kills ticks on the bitten area?
Q19	Should people wear protective clothing and apply insect repellent when in rural and woody areas?
Q20	What are the symptoms and signs of CCHF? (yes/no): Fever, dysuria, headache, hemorrhage, nausea/vomiting, constipation, diarrhea, weakness, diffuse muscle pain, anorexia, cough, rhinorrhea, low white blood cell (WBC) high WBC, anemia, vitamin B12 deficiency, high CK, low platelets, high AST/ALT, positive blood cultures, positive urine cultures.
Q21	What are the transmission methods of CCHF? a. Bite of infected tick/ b. Contact with blood, tissue or secretions of infected animals/ c. Contact with sick people’s blood or secretions/ d. Inhalation/ e. Eating the meat of animals that have been bitten by infected ticks.
Q22	Who is at risk for CCHF? a. People living in rural areas and their families/ b. Crop farmers and their families/ c. Livestock farmers and their families/ d. Soldiers/ e. Campers/ f. Scouts/ g. Wood workers/ h. People who visit rural and woody areas/ i. Butchers/ j. Abattoir workers/ k. Veterinarians.
Q23	Can CCHF be transmitted nosocomially?
Q24	Should health care workers undergo daily check-ups (of temperature and other symptoms) for 14 days after contact with infected blood and secretions?
Q25	Do you know of an effective and safe vaccine for humans against CCHF?
Q26	Has treating a CCHF patient created risk for you at your job?
Q27	Are you afraid of CCHF, although you are well informed about how it is transmitted?
Q28	Would you prefer not to work with CCFH patients if you could?

The participants’ answers were recorded.

### Statistical analyses

STATA 11.0 (College Station, TX, USA) was used for statistical analyses. The data are reported in terms of percentages. Comparisons of answers given by doctors, nurses and paramedics were performed with the program used for statistical evaluation. Data were considered with percentage calculation. Comparison of doctors, nurses and paramadics answers were performed with Chi-square test or Fisher’s exact test, as appropriate. Values of P < 0.05 were considered statistically significant.

## Results

This study included 37 (33.9%) males and 72 (66.1%) females, for a total of 109 ES workers. Mean age was 29.6 ± 6.5 (range 19 - 45). Fifty-four (49.54%) participants were doctors, 39 (35.78%) were nurses and 16 (14.68%) were paramedics. All of the participants (100%) had heard of CCHF.

Eighty-six (78.9%) of 109 participants answered that they believed they had adequate knowledge of CCHF. The general knowledge distribution of the HCWs regarding CCHF is shown in Table 2.

**Table 2:** General Knowledge Distribution of HCWs About CCHF

Question	Answer	Doctors		Nurses		Paramedics		P	Total	
		n	%	n	%	n	%		n	%
Q1	Yes	54	100	39	100	16	100	-	109	100
Q2	Yes	44	81.5	28	71.8	14	87.5	0.361	86	78.9
Q3	Yes	24	44.4	22	56.4	2	12.5	0.009	48	44
Q4	a	30	55.6	14	35.9	7	43.8	0.167	51	46.8
	b	0	0	14	35.9	0	0	< 0.001	14	35.9
	c	10	18.5	19	48.7	10	62.5	0.001	39	35.8
	d	10	18.5	18	46.2	6	37.5	0.015	34	31.2

Thirty-three (61.1%) doctors and 30 (76.9%) nurses declared that they used protective equipment (masks, gloves, and so on) during interventions for patients who were admitted to the ES with active hemorrhage. Thirty (64.8%) doctors, nine (23.1%) nurses and five (21.2%) paramedics were unaware that 1/10 diluted bleach is sufficient for disinfecting environments that are contaminated with the blood and secretions of a patient with suspected CCHF. Seventeen (40.7%) doctors, 22 (56.4%) nurses and 11 (69.7%) paramedics did not know that CCHF may be asymptomatic. Seventeen (15.6%) participants did not know that adhered ticks on the human body should not be removed by pouring a substance that kills ticks on the bitten area. The knowledge level distributions of these HCWs in terms of transmission prevention and the approach to CCHF patients are shown in Table 3. Seventy (64.2%) HCWs said that positive blood cultures are a laboratory finding in CCHF. Details regarding the HCWs’ answers regarding the symptoms and laboratory findings of CCHF are provided in Table 4. Fifty (45.9%) of the participants said that CCHF can be transmitted by inhalation. The HCWs’ knowledge level distribution regarding methods of transmission and populations at risk for CCHF is detailed in Table 5.

**Table 3:** Distribution of Participants Who Answered “Yes” to Questions About Prevention of Transmission and Approach to Patients

Question	Doctors		Nurses		Paramedics		P	Total	
	n	%	n	%	n	%		n	%
Q5	49	90.7	39	100	16	100	0.069	104	95.4
Q6	33	61.1	35	89.7	16	100	< 0.001	84	77.1
Q7	33	61.1	30	76.9	16	100	0.003	79	72.5
Q8	49	90.7	39	100	16	100	0.069	104	95.4
Q9	47	87	29	74.4	11	68.8	0.158	87	79.8
Q10a	35	64.8	37	94.9	11	68.8	0.001	83	76.2
Q10b	52	96.3	39	100	16	100	0.642	107	98.2
Q10c	54	100	39	100	16	100	-	109	100
Q10d	54	100	39	100	16	100	-	109	100
Q10e	34	63	33	84.6	9	56.3	0.036	76	69.7
Q10f	19	35.2	30	76.9	11	68.8	< 0.001	60	55.1
Q11	54	100	37	94.9	16	100	0.252	107	98.2
Q12	50	92.6	39	100	16	100	0.200	105	96.3
Q13	54	100	37	94.9	16	100	0.252	107	98.2
Q14	11	20.4	11	28.2	5	31.3	0.558	27	24.8
Q15	54	100	37	94.9	16	100	0.252	107	98.2
Q16	54	100	37	94.9	16	100	0.252	107	98.2
Q17	32	59.3	17	43.6	5	31.3	0.094	54	49.5
Q18	5	9.3	7	18	5	31.3	0.091	17	15.6
Q19	54	100	39	100	16	100	-	109	100

**Table 4:** Distribution of Participants Who Answered “Yes” to Questions About Symptoms and Laboratory Findings of CCHF (Q20)

Question	Doctors		Nurses		Paramedics		P	Total	
	n	%	n	%	n	%		n	%
High fever	54	100	39	100	16	100	-	109	100
Dysuria	10	18.5	10	25.6	2	12.5	0.516	22	20.2
Headache	52	96.3	30	76.9	9	56.3	< 0.001	91	83.5
Hemorrhage	54	100	33	84.6	16	100	0.004	103	94.5
Nausea/vomiting	51	94.4	37	94.9	16	100	1.000	104	95.4
Constipation	0	0	8	20.5	0	0	< 0.001	8	7.3
Diarrhea	37	68.5	32	82.1	16	100	0.013	85	78
Weakness	54	100	39	100	16	100	-	109	100
Diffuse muscle pain	51	94.4	33	84.6	16	100	0.136	100	91.7
Anerexia	48	88.9	37	94.8	11	68.8	0.034	96	88.1
Cough	22	40.7	7	18	5	31.3	0.065	34	31.2
Rinorrhea	24	44.4	16	41	12	75	0.058	52	47.7
Low WBC	32	59.3	27	69.2	2	12.5	< 0.001	61	56
High WBC	27	50	19	48.7	9	56.3	0.875	55	50.5
Vit B12 deficiency	2	3.7	17	43.6	5	31.3	< 0.001	24	22
High CK level	40	74.1	27	69.2	11	68.8	0.846	78	71.6
High LDH	39	72.2	27	69.2	9	56.3	0.479	75	68.8
Low thrombocytes	51	94.4	25	64.1	6	37.5	< 0.001	82	75.2
High AST/ALT	54	100	36	92.3	14	87.5	0.026	104	95.4
Positive blood cultures	34	63	25	64.1	11	68.8	0.914	70	64.2
Positive urine cultures	18	33.3	21	53.9	7	43.8	0.141	46	42.2

**Table 5:** Knowledge Level Distribution of HCWs Regarding Transmission Methods and At-Risk Populations for CCHF

Question	Answer	Doctors		Nurses		Paramedics		P	Total	
		n	%	n	%	n	%		n	%
Q21	a	54	100	39	100	16	100	-	109	100
	b	46	85.2	30	76.9	14	87.5	0.577	90	82.6
	c	49	90.7	37	94.9	16	0	0.655	102	93.6
	d	25	46.3	11	28.2	14	87.5	< 0.001	50	45.9
	e	7	13	9	23.1	7	43.8	0.028	23	21.1
Q22	a	54	100	39	100	16	100	-	109	100
	b	47	87	39	100	16	100	0.033	102	93.6
	c	54	100	39	100	16	100	-	109	100
	d	28	51.9	21	53.9	11	68.8	0.482	60	55.1
	e	54	100	37	94.9	16	100	0.252	107	98.2
	f	49	90.7	34	87.2	16	100	0.438	99	90.8
	g	52	96.3	37	94.9	16	100	1.000	105	96.3
	h	51	94.4	36	92.3	16	100	0.739	103	94.5
	i	34	63	25	64	7	43.8	0.354	66	60.6
	j	47	87	29	74.4	11	68.8	0.158	87	79.8
	k	50	92.6	34	87.2	16	100	0.347	100	91.7

Ten (9.2%) participants did not think that CCHF was associated with a transmission risk for hospital-borne infections. One hundred (91.7%) HCWs believed that caring for a CCHF patient created risk at their job, and 62 (56.9%) declared that they would prefer not to work with CCHF patients if that were an option. The personnel approach distribution of HCWs (as hospital workers) for CCHF is displayed in Table 6.

**Table 6:** Distribution of Participants Who Answered “Yes” to Questions About the Approach to CCHF as a Hospital Worker

Question	Doctors		Nurses		Paramedics		P	Total	
	n	%	n	%	n	%		n	%
Q23	51	94.4	32	82	16	100	0.057	99	90.8
Q24	51	94.4	36	92.3	16	100	0.739	103	94.5
Q25	52	96.3	39	100	16	100	0.642	107	98.2
Q26	47	87	37	94.9	16	100	0.305	100	91.7
Q27	46	85.2	32	85	16	100	0.204	94	86.2
Q28	36	66.7	15	38.5	11	68.8	0.015	62	56.9

Among 86 (78.9%) HCWs who believed that their knowledge about CCHF was sufficient, 50 (58.1%) declared that they would prefer not to follow patients with CCHF (P = 0.608).

## Discussion

HCWs are an important risk group for CCHF infection in endemic areas. Infected patients should be isolated, and barrier nursing techniques should be used. Strict universal precautions are necessary, and health care workers should wear protective clothing such as disposable gowns, gloves and masks, as well as goggles or face shields. During procedures that may produce aerosols, an N95 mask should be worn. Human infections are mainly caused by direct contact with blood or tissues of viremic hosts, as well as by tick bites or crushing infected ticks with unprotected hands. In endemic areas, high-risk groups include persons who have occupational contact with livestock and other animals, such as farmers, livestock owners, abattoir workers and veterinarians. Recreational activities such as hiking and camping in endemic areas are also risk factors for tick bites. As the CCHF virus is destroyed by tissue acidification and does not survive cooking, meat consumption is safe. The ratio of subclinical to clinical CCHF cases is approximately 5:1, and 80% of infections are asymptomatic. The nosocomial route is an important transmission mechanism for CCHF. HCWs caring for patients with CCHF are a major risk group. Direct transmission is thought to occur through contact of broken skin with viremic blood or other body fluids. Interventions for gastrointestinal bleeding, surgery on patients with occult disease, needle stick injuries and unprotected handling of infected materials are high-risk activities. Case fatality rates among nosocomial cases tend to be higher than in community-acquired cases, which may be related to the viral inoculums [15].

Emergency room HCWs constitute a high-risk group for blood- and secretion-borne infections [9]. For many emergency room patients, it is often difficult to obtain a detailed medical history because of time constraints. For example, when a patient is admitted to the emergency room with hemorrhage, contact with the patient begins before laboratory evaluations can be obtained. Therefore, emergency room physicians, nurses and other HCWs must begin care before having definitive information about a patient’s previous health history and current diagnosis. Despite these complicating factors, HCWs are responsible for protecting themselves against infectious diseases. Therefore, knowledge of infectious diseases and their transmission methods, especially in endemic regions, is important for HCWs. A young emergency resident physician died in October 2012 due to a needle stick injury while caring for a CCHF patient in Turkey [16]. There are a limited number of studies regarding CCHF knowledge levels among HCWs in the literature. In this study, we aimed to investigate CCHF knowledge levels among emergency department HCWs in an endemic region.

In Rahnavardi et al’s cross-sectional study, 209 HCWs from three hospitals in a region where CCHF was common were included. In this study, 11 (5.8%) participants had heard of CCHF. In our study, all of the participants (100%) had heard about CCHF. These findings suggested that being a physician and relying on academic material rather than local media were independently and significantly associated with higher knowledge levels. Education levels and laboratory staff attitudes were also significant factors. Forty-four percent of the study group wore gloves and masks for contact with CCHF patients, and 22% failed to observe any safety measures [17]. In our study, 86 (78.9%) of 109 participants believed that their knowledge levels regarding CCHF were sufficient.

Fifty-four (49.54%) participants were doctors, 39 (35.78%) were nurses and 16 (14.68%) were paramedics. Thirty-three (61.1%) doctors and 30 (76.9%) nurses declared that they used protective equipment (masks, gloves, and so on) during interventions for patients who were admitted to the emergency department with active hemorrhage. Thirty (64.8%) doctors, nine (23.1%) nurses and five (21.2%) paramedics were unaware that 1/10 diluted bleach is adequate for disinfecting environments that are contaminated with the blood and secretions of a suspected CCHF patient. Seventeen (40.7%) doctors, 22 (56.4%) nurses and 11 (69.7%) paramedics were unaware that CCHF may be asymptomatic. Seventy (64.2%) HCWs said that positive blood cultures are a laboratory finding in CCHF. Fifty (45.9%) of the participants said that CCHF can be transmitted by inhalation. Paramedics were more compliant than doctors and nurses with preventative measures.

Ten (9.2%) participants did not think that CCHF could be transmitted nosocomially. One hundred (91.7%) HCWs believed that caring for a CCHF patient created workplace risk, and 62 (56.9%) declared that they would prefer not to work with CCFH patients if possible.

Yilmaz et al attempted to determine knowledge levels, attitudes and practices regarding CCHF in people visiting a tertiary care hospital in an endemic city in Turkey. They provided questionnaires to the relatives or guardians of patients who were admitted to pediatric outpatient clinics and studied 1,034 participants. According to these authors, the media are the most useful source of information on this disease. They also described insufficient knowledge regarding CCHF in the normal population and suggested that the health, agriculture and media sectors can improve public knowledge and awareness of CCHF [18].

### Conclusions

In the 10th year after the first CCHF outbreaks in Turkey, we demonstrate that ES HCWs in endemic regions have insufficient knowledge about this disease. We believe that seminars and education about CCHF and its transmission methods may be helpful for ES HCWs; furthermore, undergraduate curricula for all health-related courses should be reviewed to ensure effective education on this topic. Most CCHF patients first present in the emergency room. Therefore, techniques that protect against transmission of this disease, including gloves (especially baricidal gloves), face masks, face visors and box coats, should be explained to ES HCWs, and the use of these techniques should be encouraged.

This report describes the first study of CCHF knowledge levels among emergency room HCWs in an endemic region. In the future, comprehensive studies may be helpful to prevent the deaths of HCWs due to this disease.
